# An Experimental Method to Capture the Thermal Conductivity Coefficient of Fine-Grained Concretes during Transition from Liquid to Solid

**DOI:** 10.3390/ma17092115

**Published:** 2024-04-30

**Authors:** Yannik Schwarz, Denis Ratke, David Sanio, Thomas Meurer, Peter Mark

**Affiliations:** 1 Institute of Concrete Structures, Ruhr-Universität Bochum, 44780 Bochum, Germany; david.sanio@rub.de (D.S.); peter.mark@rub.de (P.M.); 2 Digital Process Engineering, Karlsruher Institut für Technologie, 76131 Karlsruhe, Germany; denis.ratke@kit.de (D.R.); thomas.meurer@kit.de (T.M.)

**Keywords:** temperature induction, thermal conductivity coefficient, high-performance concrete, hydration-dependent thermal conductivity coefficient, digital twin

## Abstract

During the transition from liquid to solid, the thermal conductivity coefficient λ of concrete decreases. Although λ of hardened concrete is well investigated, there is limited research on the transition from liquid to solid and how it depends on hydration. Currently, only simplified qualitative approaches exist for the liquid state and the transient process. An experimental method is not available. For this purpose, a test rig is designed to experimentally capture the evolution of λ for fine-grain concretes during transition. The performance of the test setup is evaluated on a characteristic high-performance concrete (HPC). The results are compared to theoretical predictions from the literature. The developed test rig is mapped in a digital twin to investigate extended boundary conditions, such as different heat sources and temperatures of the experimental setup. It allows the experiment to be repeated and optimized for different setups with little effort. The test principle is as follows: A liquid concrete sample is heated through a controlled external source, while the transient temperature distribution over the height is measured with a fiber optic sensor. The thermal conductivity is derived from the heat flux induced and the temperature distribution over an evaluation length. Experiments show that *λ* in the liquid state is approximately 1.4 times greater than in the solid state and exponentially decreases for the transient process. Numerical results on the digital twin indicate that the robustness of the results increases with the temperature of the heat source. Moreover, the derivation in λ turns out to be strongly dependent on the evaluation length. A length of three times the maximum grain diameter is recommended.

## 1. Introduction

The heat transfer in solid materials is driven by the thermal conductivity coefficient *λ* [1]. Since *λ* depends on hydration [2], it decreases for young concrete during solidification, which is the transition from liquid to solid. Although being an important aspect of thermal material behavior, the transient course of *λ* during transition has not been thoroughly studied. The change in λ is particularly relevant during the heat treatment of young concrete, as it accelerates solidification and, thus, the transition of λ. The heat treatment of young concrete has become an integral part of materials technology. It is used, among other things, to accelerate strength development or reduce the long-term deformations of creep and shrinkage [2,3,4]. Knowledge of the transient course of *λ* is relevant to numerically modeling and optimizing the heat treatment process. While *λ* for hardened concrete is well known, only simplified qualitative approaches exist for the liquid state and the transition in between [5]. Currently, no experimental method is available to determine *λ* during hydration.

The thermal conductivity of hardened concrete depends on composition [6,7]. In particular, the water/cement ratio (*w*/*c*), the aggregates, the fines, and the type and quantity of admixtures are relevant [6,7]. Aerated concrete exhibits generally lower thermal conductivity due to the insulating properties of the air [8].

Table 1 shows ranges of *λ* from the literature for various concrete categories [1,9]. Only a few studies are available for HPC and ultra-high-performance concrete (UHPC), so specific ranges cannot be reported; only rough reference values are indicated.

Even for the same composition, the thermal conductivity of concrete depends on its temperature and moisture content [6,7]. The investigations in [10] on the influence of temperature on *λ* show that the thermal conductivity can be assumed to be constant at temperatures up to 100 °C. At higher temperature much greater than 100 °C, as in the case of fire, *λ* decreases significantly [10].

Under ambient temperature conditions, the moisture content becomes more relevant [7,11] since the thermal conductivity of water is around 25 times greater than that of air [7,11]. Therefore, the thermal conductivity of concrete increases with the moisture content and consequently decreases with the air void content. During hydration, water is chemically bonded; therefore, *λ* decreases [5].

In the literature, only simplified qualitative approaches for the hydration dependence of *λ* are proposed. In ref. [5], a linear approach is presented with respect to the degree of hydration *α* (Equation (1)). Therein, *λ*_0_ is the initial thermal conductivity of the freshly mixed concrete in the liquid state, while *λ*_hard_ represents the fully hydrated state. The parameter *α* is defined as the ratio of heat released up to the considered time to the total hydration heat quantity with 0 ≤ *α* ≤ 1 [5,12].

(1) $$\lambda \left(\alpha \right)=\lambda_{0}-(\lambda_{0}-\lambda_{hard})\alpha$$

To determine the initial conductivity $\lambda_{0}$, in [5], an approach according to Kirchner [13] is used (Equation (2)), which depends on the volume-related moisture excess ϕ (Equation (3)). This indicates the proportion of the water that is not chemically bound by the cement during mixing; in normal concrete, it amounts to about 75% [5,13]. Then, ϕ can be calculated for a specific concrete as a function of the cement *c* and the water *w* contents.

(2) $$\lambda \left(\phi \right)=\lambda_{hard}\cdot \frac{1+12\phi }{1.6}$$

with

(3) ϕ=(w−0.25c)/1000

Feeding ϕ from Equation (3) into Equation (2), *λ*_0_ can be calculated in relation to *λ*_hard_.

(4) $$\lambda_{0}=\frac{\lambda_{hard}}{1.6}\left(1+\frac{12w-3c}{1000}\right)$$

According to [5], and depending on the *w*/*c* ratio, *λ*_0_ is 30% to 55% greater than *λ*_hard_. However, the evolution of *λ* during hydration is not considered in detail, and the approach is limited to normal concrete.

Experimental methods to determine *λ* for solids can be divided into steady-state and transient approaches [14]. The guarded hot-plate measurement [15,16,17] and the heat-flow meter [18,19] are the most commonly used steady-state methods for solid materials. In the associated tests, a material sample is heated until a stationary temperature distribution is reached within. Then, the *λ*-value is derived from the sample’s geometry, the heat flux induced, and the temperature gradient over a defined length. In particular, for materials with low thermal conductivity, such as concrete, it may take several hours to reach stationarity. At this time, the concrete is already almost fully hydrated. So, the evolution of *λ* during solidification cannot be determined with these methods.

Transient methods have the advantage of generating results in a short time. The hot-wire method is most commonly used [20,21]. A material sample is heated using a known power-immersed heating wire; *λ* is then derived from the temperature rising with time at a certain distance from the wire. An alternative is the laser flash method [22,23]. A material sample is heated with a laser pulse on one side, and the temperature increase is recorded on the opposite side using an infrared thermometer. Then, *λ* is determined from the sample thickness and the time elapsed between the laser pulse and the temperature increase on the opposite side. However, transient methods have limitations in determining *λ* during hydration. The material sample must maintain a constant temperature, allowing for repeated measurements only after complete cooling. This means that changes in material properties can only be recorded over long-time intervals. Additionally, transient methods are less accurate than stationary methods and are most suitable for small material samples with dimensions of a few millimeters only [24].

To fill this gap, a test rig is designed to determine the evolution of *λ* for fine-grain concretes during solidification from experiments.

For this, the contribution is structured as follows: Section 2 presents the method developed to experimentally derive *λ* during hydration in general and the implementation of the test rig in a digital twin. The numerical model represents the thermal behavior of concrete during hydration and is calibrated with the test data.

In Section 3, the test rig is verified in an experiment on a characteristic HPC. The results are compared with the numerical ones obtained on the digital twin. The digital twin is utilized to assess the suitability of the test rig for concretes with other boundary conditions (Section 4).

## 2. Experimental Determination of the Transient Thermal Conductivity Coefficient

### 2.1. Theoretical Basis for the Development of the Test Rig

If heat is supplied to a system, the temperature in the system balances with time due to the heat transfer. This comprises thermal conduction, radiation, and convection [1]. Heat transfer in solids is limited to thermal conduction. For homogeneous and isotropic materials, the heat flux $\dot{Q}$ can be calculated with Fourier’s law (cf. [1,25]). In case of one-dimensional heat conduction, $\dot{Q}$ depends on the thermal conductivity of the body, the cross-sectional area *A* of the heat flux, and the temperature gradient dϑ over the length dx (Equation (5)). *λ* is a scalar and describes the material’s ability to transfer heat [W/(mK)]. In general, it is considered as a constant material property.

(5) $$\dot{Q}=-\lambda \cdot A\cdot \frac{d\vartheta }{dx}$$

$\dot{Q}$ in the interval [*x*_1_, *x*_2_] is determined as follows:

(6) $$\int_{x_{1}}^{x_{2}}\dot{Q}\cdot dx=\int_{\vartheta_{1}}^{\vartheta_{2}}-\lambda \cdot A\cdot d\vartheta$$

Assuming a linear temperature gradient in the interval [*x*_1_, *x*_2_] with $∆x=x_{2}-x_{1}$ and $∆\vartheta =\vartheta_{1}-\vartheta_{2}$ Equation (6) can be written as follows:

(7) $$\dot{Q}=\frac{\lambda }{x_{2}-x_{1}}\cdot A\cdot \left(\vartheta_{1}-\vartheta_{2}\right)=\lambda \cdot \frac{A\cdot \Delta \vartheta }{\Delta x}$$

Solving for *λ* gives

(8) $$\lambda =\frac{\dot{Q}\cdot ∆x}{∆\vartheta \cdot A}$$

In what follows, Equation (8) is used to derive *λ* for homogeneous and isotropic materials from the tests. Therefore, a linear temperature gradient in the interval ∆x is required.

### 2.2. Design of the Test Rig

**Figure 1:**
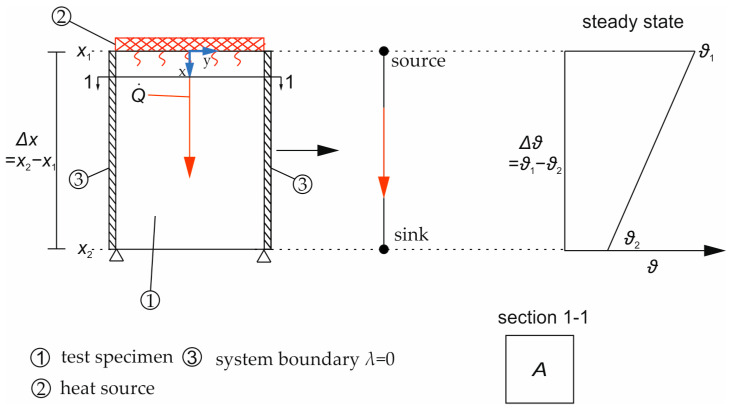
Test principle for determination of the thermal conductivity coefficient of solid materials and others with heat transfer just through thermal conduction.

The idea of the test rig (Figure 1) is to generate a uniaxial linear temperature distribution in a test specimen (1) between an external heat source on top (2) and a free surface (sink) on the bottom. The sides are thermally insulated (3). The heat supply and the temperature distribution in the specimen are both measured continuously.

For evaluation, the setup is interpreted as a 1D system, as shown in Figure 1 at the center, with a linear temperature distribution in the steady state between the source and the sink (right). To derive *λ* for the tested material, the temperature gradient ∆ϑ, over the length ∆x (here, ∆x = specimen height), and the heat flux $\dot{Q}$ from the external heat source induced over the cross-sectional area *A* are substituted into Equation (8).

### 2.3. Evaluation of the Thermal Conductivity Coefficient

**Figure 2:**
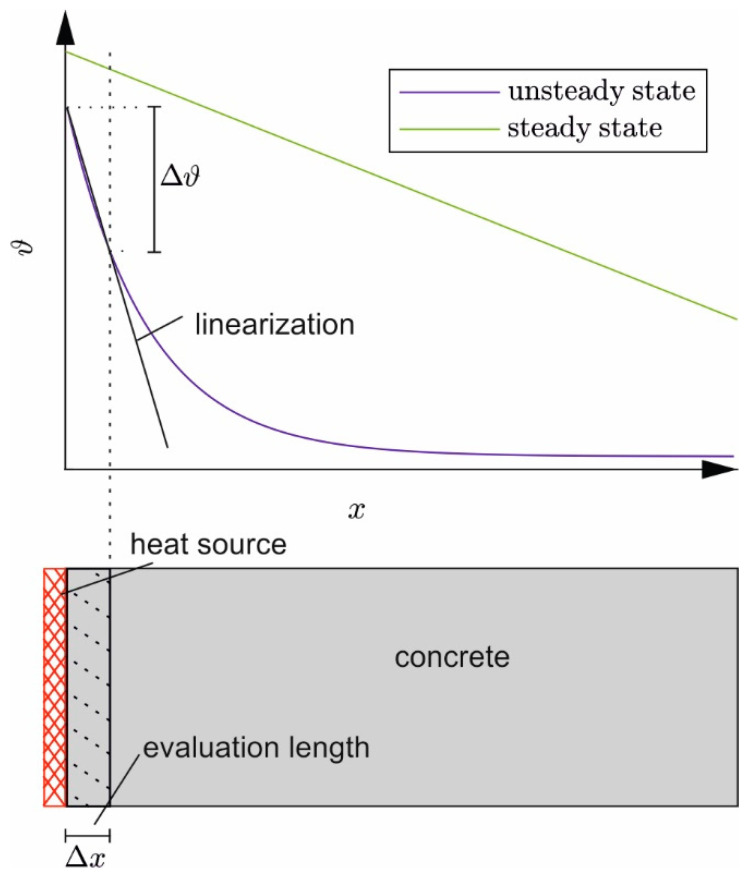
Temperature distributions in the specimen for the transient and steady-state conditions (**top**)—reduced evaluation length in the layer for an approximately linear temperature distribution (evaluation length, **bottom**).

The test-based evaluation of *λ* according to Equation (8) requires a linear temperature distribution over ∆x. Figure 2 sketches the temperature distribution in the specimen for the steady state and when heating just has started (unsteady state). Initially, the temperature only increases near the heat source; deeper layers do not yet experience a rise in temperature—an almost linear temperature gradient only develops in the boundary layer near the heat source. To derive *λ* at this early stage, the evaluation length ∆x needs to be limited to a near-surface layer.

From this and just with time, an approximately linear temperature gradient forms over the entire sample length. Then, ∆ϑ can be evaluated on the total height of the specimen.

### 2.4. Digital Twin

Along with the experimental investigations, a digital model is developed to analyze the thermochemical process of concrete heating and solidification. This development includes the identification of a mathematical representation of the system, its numerical solution, and parametrization. The solidification process is described by a set of three (coupled) partial differential equations (PDEs)

(9) $$\;\;\;\;\;\rho c_{p}\partial_{t}T=\partial_{x}\left(\lambda \left(m\right)\partial_{x}T\right)+\partial_{t}Q,$$

(10) $$\partial_{t}\theta =\partial_{x}\left(D\left(\theta \right)\partial_{x}\theta \right)-\eta \partial_{t}m,$$

(11) $$\;\;\;\;\;\;\;\;\partial_{t}m=\mu \left(1-m\right)\theta e^{-E/RT},$$

which are defined on the domain $\Omega =\left(x,t\right):x\in \left(0,L\right),t\in (0,\tau )$ [26]. Herein, $T\left(x,t\right)$ describes the evolution of the temperature in [K] (while ϑ is the temperature in [°C]), and $\theta \left(x,t\right)$ is the (relative) moisture. To represent the degree of hydration, the maturity $m\left(x,t\right)$ is introduced. It should be noted that only the progression in the *x*-direction is considered due to symmetry considerations. The thermal characteristics are defined by means of heat capacity $c_{p}$, conductivity $\lambda \left(m\right)$ and material density ρ. The generated heat caused by hydration is given by the source term.

(12) $$\partial_{t}Q=Q_{m}\partial_{t}m,\;Q_{m}=Ame^{-am^{2}},$$

where $a=1/2m_{x}^{2}$ and $A=Q_{x}/m_{x}$, with $m_{x}$ indicating the specific *m* for which $Q_{m}$ reaches its maximum $Q_{x}$. Equation (13), with $D_{m}$ as moisture diffusivity coefficient, reflects the nonlinear behavior of moisture diffusion in the concrete according to [26].

(13) $$D\left(\theta \right)=D_{m}\left[0.05+0.95\left(1+\tanh\left(20\left(\theta -0.8\right)\right)\right)\right]$$

The sink term $\eta \partial_{t}m$ in the moisture formula (Equations (9)–(11)) is the hydration reaction ratio η. The activation energy, gas-constant, and reaction rate are given by *E*, *R*, and μ. The required initial

(14) $$T\left(x,0\right)=T_{0}\left(x\right),\;\theta \left(x,0\right)=1,\;m\left(x,0\right)=0$$

and boundary conditions

(15) $$-{\lambda \left(m\right)\partial }_{x}T{|}_{x=0}=\sigma_{1}\left(T\left(x,t\right)-T_{s,\;\infty }\right),\;{\lambda \left(m\right)\partial }_{x}T{|}_{x=L}=\sigma_{2}\left(T\left(x,t\right)-T_{m,\;\infty }\right)+\dot{q},\;-D\left(\theta \right)\partial_{x}\theta {|}_{x=0}=\bar{e}\left(\theta_{a}-\theta \left(L,t\right)\right),\;D\left(\theta \right)\partial_{x}\theta {|}_{x=L}=0,$$

for the PDEs in Equations (9)–(11) result from the experimental setup and the assumptions that $m\left(x,t\right)\in \left[0,\;1\right],\;\theta \left(x,t\right)\in \left[0,\;1\right]$. Here, $\bar{e}$ is the evaporation rate, and the heat transfer coefficients—assumed as constants—are denoted as $\sigma_{1}$ and $\sigma_{2}$; $T_{s,\;\infty }$ refers to the temperature of the steel plate at x=0. The measured values of the heat mat temperature and heat flow induced through it at position x=L  can be referred to as $T_{m,\;\infty }$ and $\dot{q}$, respectively. The experimental configuration incorporates a heat flux sensor and a protective foil between the heat mat and the concrete surface, preventing any direct contact (see Section 3.1). A combination of the heat flux and the Robin boundary condition, in which the temperature of the mat is a contributing factor, is utilized as the energy input into the concrete to make up for the absent energy input at the position x=L.

The parameter identification process was conducted to determine the values of $c_{p}$, μ, $D_{m}$, η, $m_{x}$, $Q_{x}$, $\sigma_{1}$, $\sigma_{2}$, $T_{1,\;\infty }$, and $T_{2,\;\infty }$, as well as the functional relationship of the conductivity $\lambda \left(m\right)$. Further parameters were taken from [26]. The parameters from the literature and the identified ones are summarized in Table 2.

The system parameterization was performed using MATLAB, aiming to minimize the magnitude of the Euclidean distance between the measured and numerically calculated temperatures. Note that, currently, a measurement of moisture and maturity is not available in the experiment. Additionally, for the determination of the thermal conductivity $\lambda \left(m\right)$ different ansatz functions were examined to express the dependency of *λ* on maturity, eventually leading to the following formulation:

(16) $$\lambda \left(m\right)=\lambda_{0}\left(1+e^{-m}\right)$$

with $\lambda_{0}$ adjusted to reduce the distance to the experimental results. The dependency on maturity reflects the changes in thermal conductivity during the solidification process.

**Figure 3:**
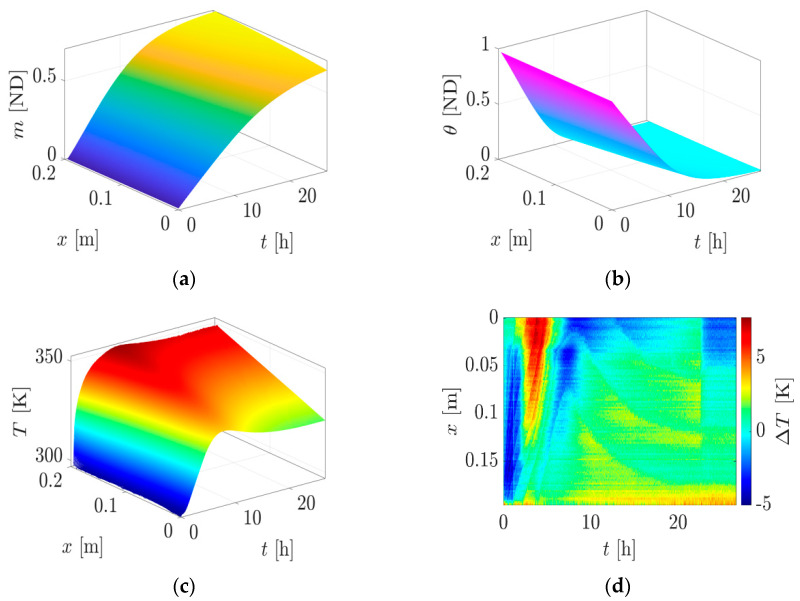
Numerical solution of Equations (9)–(11) for the parameters of Table 2 and the error between measured and numerically calculated temperature: (**a**) evolution of maturity; (**b**) evolution of moisture; (**c**) evolution of temperature; (**d**) error between the measured and numerically calculated temperature.

Given the inherent complexity of the equations involved, obtaining an analytical solution is impractical in this scenario. Instead, the solution is approximated numerically using the finite-difference method on a uniform grid, as exemplified in [27]. The numerical simulation of the discrete system with the parameters from Table 2 and the difference between the measured and numerically calculated temperatures are illustrated in Figure 3. Comparing Figure 3a,b, which reflect the development of the maturity and moisture, it is easy to see that both behave oppositely. During hydration, the water is bound, and the moisture content decreases. The influence of hydration on temperature behavior can be identified as a bulge, as shown in the first ten hours of the temperature course in Figure 3c. The error between the measured and numerically simulated temperature is given in Figure 3d. The maximum deviation along the simulation and measurement occurs in the first hours, where also the most intense dynamics of the heating process occur.

## 3. Experiments

### 3.1. Experimental Setup

The developed test rig was exemplarily evaluated on a characteristic HPC. The tested concrete is based on a nanotechnologically optimized binder and has a compressive strength $f_{c}>125$ N/mm^2^ [9,28]. The HPC is suitable for rapid heat treatment without pre-storage and high temperatures (<100 °C). The investigations in [29] show rapid strength development without any relevant damage influences due to secondary ettringite formation. Table 3 shows the concrete composition.

**Figure 4:**
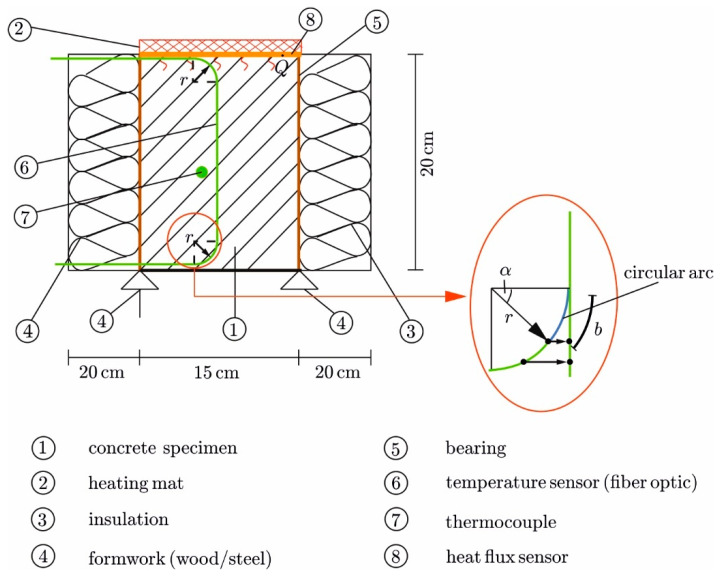
Configuration of the test rig with measurement equipment (**left**) and installation of the fiber optic sensor near the surfaces (**right**).

**Figure 5:**
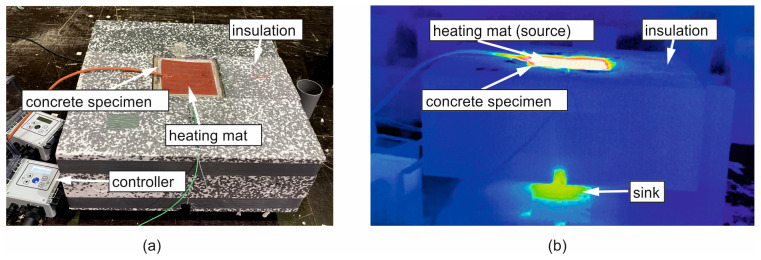
Test setup (**a**) and a thermal image during heating (**b**); in both photos, the upper insulation is removed.

The principle of the test rig according to Section 2.2 is given in Figure 4, with the implementation presented in Figure 5. The specimen had the dimensions *h*/*w*/*l* = 20/15/15 cm and initially consisted of liquid concrete (Table 3), filled into a formwork. The sides of the formwork were made of wood, and a steel plate was used at the bottom (thickness *h* = 5 mm, λ = 50 W/(mK)) to ensure heat dissipation at the sink. With the formwork, the specimen was supported laterally. The insulation was realized using polystyrene boards (*h* = 20 cm, λ = 0.032 W/(mK)).

Concrete was heated by a silicon heating mat of the same size as the cross-section area. The mat had a wattage of 3000 W/m^2^ and was equipped with a two-point controller and a temperature sensor to maintain a once-set temperature constant. To concentrate $\dot{Q}$ in the direction of the concrete, insulation (*h* = 20 cm) was placed on the upper side of the mat, too. Additional weights pressed the mat against the liquid concrete to ensure full contact [30]. A heat-resistant Hostaphan foil (*h* = 0.05 mm) separated the mat from the liquid concrete.

To measure the transient temperature field, fiber optic sensors (DFOS) were laid longitudinally in capillaries in the concrete (no. 6 in Figure 4) [31]. The capillary prevents contact between the fiber and the surrounding concrete, so the fibers are not subjected to mechanical strains, allowing for the measurement of pure thermal strains [31]. The distance of the measuring points (gauges) along the fiber was 0.65 mm, and the measuring frequency was 1 Hz. Due to the heating mat on top and the steel formwork at the bottom, the DFOS could not be laid straight through the test specimen. Instead, the fiber had to be led out to the side geometrically following a circular arc (detail in Figure 4), with a 2 mm distance to the surfaces on the top and bottom. A minimum radius of *r* > 1 cm is required to bend but not destroy the fibers [32]. Here, that radius was selected as *r* = 3.5 cm. This bending of the fiber had to be compensated in the postprocessing stage to ensure the precise location of each gauge along the vertical axis (*y*). The gauges along the circular arc were converted into an equivalent straight line, as shown in Figure 4 on the right.

Relevant points along the fiber (start and end of the arcs) were marked using the hot-spot method [31]. This was accomplished by heating the points locally with a pin before concreting, which identified the spots in the data record. The angle α=b/r is the ratio of the arc length *b* to the radius *r*. For each point along the arc, the vertical distance (in *y*) from the starting point of the arc is as follows: *y*′ = cos(*α*)*r.

Temperature changes relative to an initial state were measured in the fiber. An additional thermocouple next to the fiber recorded the absolute temperature and enabled the calibration of DFOS measurements.

The time-dependent course of $\dot{Q}$ induced by the heating mat was recorded with a heat flux sensor (Hukseflux FHF04SC, *h* = 0.4 mm) placed between the concrete and the heating mat [33,34]. The measurement uncertainty was ±0.2%.

### 3.2. Experimental Procedure

The fresh concrete was filled into the formwork and tempered for 22 h with a constant temperature of the heating mat $\vartheta_{M}$ = 80 °C. This temperature limit was intentionally set to not exceed 100 °C, even when additional hydration heat emerged. If the limit is not met, the admixture water might start boiling, and structural damage must be expected. A safety margin of 20 °C was considered to compensate for hydration heat.

Figure 5a shows a photo of the experimental setup and a corresponding thermal image from infrared thermography during the experiment Figure 5b. Figure 5a shows the heating mat with concrete underneath, temperature controller, and lateral insulation. The insulation on the heating mat has been removed for the photo. The infrared thermography on Figure 5b gives an impression of the heat propagation in the test specimen and the effectiveness of the insulation. Heated areas with a temperature of about 80 °C are color-coded in red to white, while the cold regions with temperatures of about 20 °C are blue. The heat induced at the top flows through the concrete and transfers to the non-insulated bottom.

### 3.3. Results

**Figure 6:**
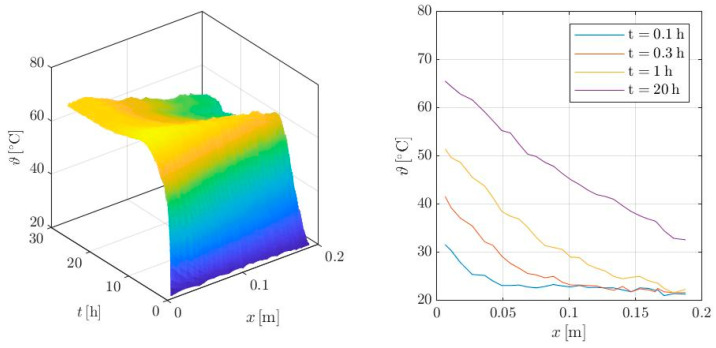
Temperature evolution in the specimen (**left**) and at discrete times (**right**) measured with fiber optic sensors.

The transient temperature distribution in the specimen as measured by DFOS is shown in Figure 6 on the left. The diagram plots the temperature development over time *t* and the specimen length *x*, with low temperatures in blue and high temperatures in orange to yellow. In the beginning, the liquid concrete has an almost constant temperature of 21 °C. Heating results in an uneven temperature increase in the specimen, beginning from the top. Moreover, tempering accelerates hydration, and the hydration heat superposes with the externally supplied heat. This effect reaches its maximum after approx. 6 h and a concrete temperature of 76 °C. The concrete temperature at the sink decreases as the hydration heat declines. After approx. 20 h, the temperature distribution becomes stationary.

Figure 6 on the right displays the temperature distribution recorded over the sample height at discrete time points. After 0.1 h of heating, the temperature distribution in the boundary layer (*x* ≤ 0.04 m) is almost linear. The deeper layers do not yet exhibit any significant temperature increase. With continued heating, the length of the almost linear distribution increases. The maximum temperature in the stationary state is approx. 68 °C. This is attributed to the measurement distance of 2 mm from the concrete surface and the unavoidable unevenness of the tempered concrete surface with corresponding losses [4].

**Figure 7:**
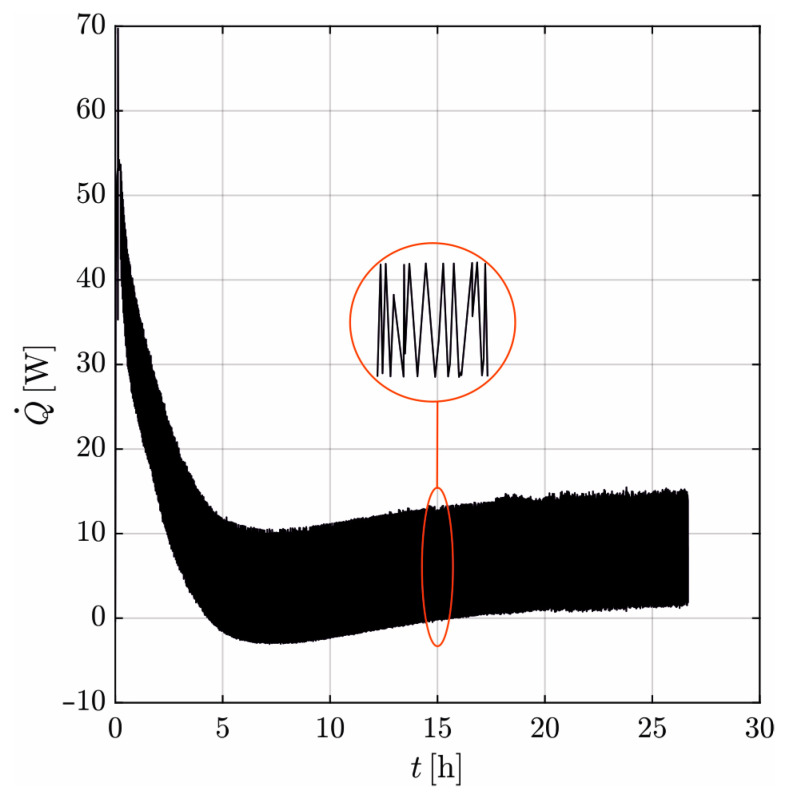
Heat flux over time recorded with the heat flux sensor.

Figure 7 shows the induced $\dot{Q}$ over time recorded with the heat flux sensor. The heating mat induces ${\dot{Q}}_{max}$ = 55 W in the beginning. Meeting the set temperature of 80 °C, the controller of the heating mat keeps it constant by switching “On” and “Off” selectively as soon as the measured temperature deviates from the wanted temperature by 0.5 °C. The heating mat induces either full or no heat, which results in an alternating course of $\dot{Q}$ in the sensor (detail in Figure 7). Since the temperature gradient between the heating mat and the concrete decreases with time, $\dot{Q}$ also decreases on a global scale. In the period 5–10 h, $\dot{Q}$ is partially negative. This can be attributed to the dissipation of the hydration heat. In the stationary state, the mean value of $\dot{Q}$ is constant at about 6 W throughout.

### 3.4. Evaluation of the Transient Thermal Conductivity Coefficient

The time-dependent evolution of *λ* is determined according to Section 2.3 and Equation (8). In the transient state, the temperature distribution is evaluated over a reduced Δx. It is necessary to determine a minimum evaluation length to ensure sufficient temperature gauges along the considered length and to assume an approximately homogeneous concrete composition. According to [35], the minimum concrete thickness for thermal evaluations should be at least three times the largest grain diameter of the concrete ($D_{max}$). Here, Δx is 9 mm.

**Figure 8:**
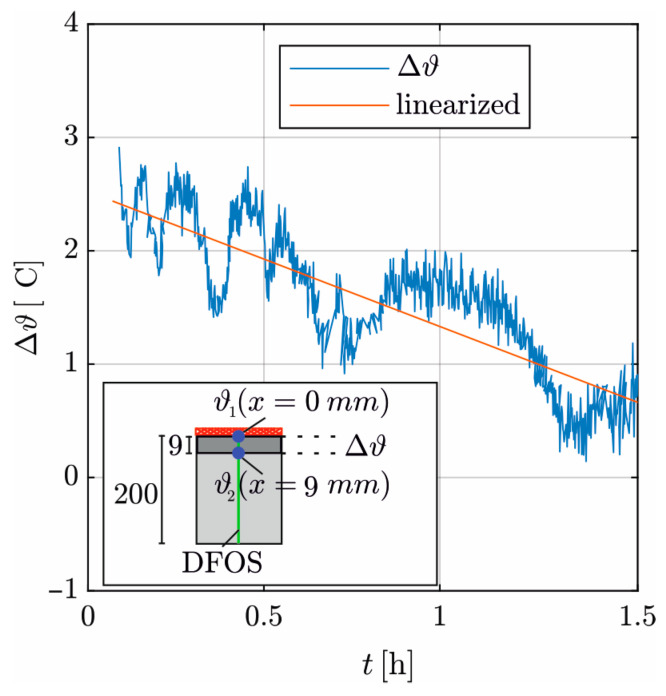
Time course of the linear temperature gradient on the evaluation length with linearization.

Figure 8 shows the time course of Δϑ over Δx derived from the DFOS measurements, with Δϑ = (ϑ(x=0mm,t)−ϑ(x=9mm,t)) in the interval 0.08 to 1.5 h. For *t* < 0.08 h, the temperature distribution over Δx is nonlinear. The alternating course of $\dot{Q}$ (Figure 7) is also found here to be attenuated in time due to the inertia of concrete heating. Therefore, the linearization of the curve by minimizing the mean-squared error is required for the evaluation.

The influence of the hydration heat increases over time until *t* = 6 h. The internally released heat flows toward both edges and thus partially in the opposite direction to the externally supplied heat, reducing Δϑ. For *t* > 1.5 h, this results in a negative Δϑ, and λ cannot be derived until the steady state is reached.

For the determination of *λ*, Equation (17) is used with the linearization of ∆ϑ(t) (Figure 8), $\dot{Q}(t)$ according to Figure 7, ∆x = 0.009 m, and *A* = 0.0225 m^2^.

(17) $$\lambda (t)=\frac{\dot{Q}(t)\cdot ∆x}{∆\vartheta (t)\cdot A}$$

**Figure 9:**
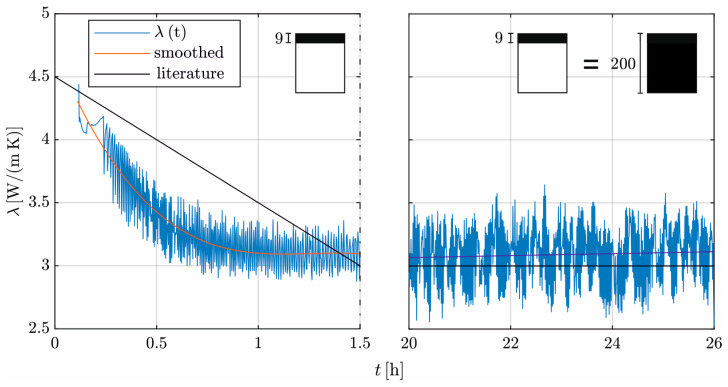
Time course of the thermal conductivity of HPC in the transition from liquid to solid with an indication of the evaluation length, compared to the literature [18].

Figure 9 shows the time-dependent development of *λ* for the investigated concrete. At *t* = 0.08 h, *λ* is 4.3 W/(mK). For *t* > 1.0 h, λ stabilizes at 3.1 W/(mK). In the transition phase, there is an exponentially decreasing course.

For *t* = 20 h, the temperature distribution in the specimen reaches a stationary state, allowing for the determination over the entire sample length (∆x = 20 cm) and resulting in *λ* = 3.06 W/(mK). This value is in accordance with the result after one hour with a reduced evaluation length.

The alternating course results from the alternation in $\dot{Q}$ and the inertia of concrete heating. In the stationary state, the heating mat has longer on/off phases to maintain a constant temperature. Furthermore, the increased evaluation length amplifies the impact of the inertia of concrete heating. Both effects result in a higher oscillation of the heat flux with a greater time gap to ∆ϑ.

## 4. Discussion

### 4.1. Comparison of the Results with Approaches from the Literature

A simplified method to determine the hydration dependence of *λ* is suggested in the literature. Table 4 gives a comparison of the theoretical and the experimental results. Due to limitations in the experiment, it is not possible to determine an exact value for *λ* before *t* = 0.08 h, but the approach $\lambda_{0}$ = (1.3…1.55) $\lambda_{hard}$ according to [5] approximates the experiment well. According to [32], the investigated concrete has a thermal conductivity of $\lambda_{hard}$ = 3.0 W/(mK). The experimentally determined coefficient thus deviates from the literature by less than 4%.

The experimental results show an exponentially decreasing slope for *λ*, which differs from the simplified linear slope according to ref. [5] in Figure 9.

### 4.2. Evaluation Length

**Figure 10:**
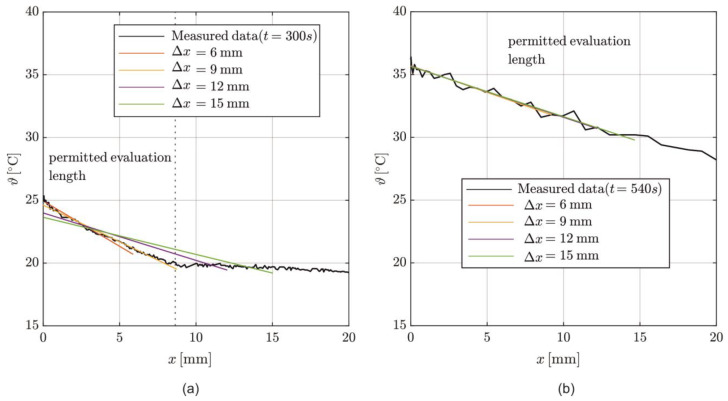
Temperature distribution in the specimen recorded by DFOS at t = 300 s (**a**) and t = 540 s (**b**) with linearized temperature gradients for different evaluation lengths **Δ*x***.

To evaluate *λ* according to Equation (8), a linear temperature distribution over Δx is necessary. Figure 10 shows the temperature distributions measured by DFOS in the interval *x* = [0, 20 mm] of the specimen for *t* = 300 s Figure 10a and *t* = 540 s Figure 10b, with the linearization of the temperature profile on varying Δx. For *t* = 300 s, the temperature only increases in the top layer (*x* ≤ 8.7 mm), as indicated by the vertical dashed line Figure 10a. The temperature distribution measurements show high consistency with the linearization for Δ*x* = 6 mm and Δ*x* = 9 mm, with determination coefficients of *R*^2^ = 0.91 and 0.95, respectively. A more extended evaluation length underestimates the temperature gradient, resulting in *R*^2^ = 0.83 and 0.74 for Δ*x* = 12 mm and 15 mm.

For *t* = 540 s Figure 10b, a linear temperature distribution is reached up to *x* = 20 mm, with a good approximation for all Δ*x* considered. The determination coefficients are all *R*^2^ ≥ 0.95.

For an accurate assessment of *λ*, a short evaluation length of 9 mm is sufficient at early times. As the tempering time increases, the permissible evaluation length also increases but is not required.

**Figure 11:**
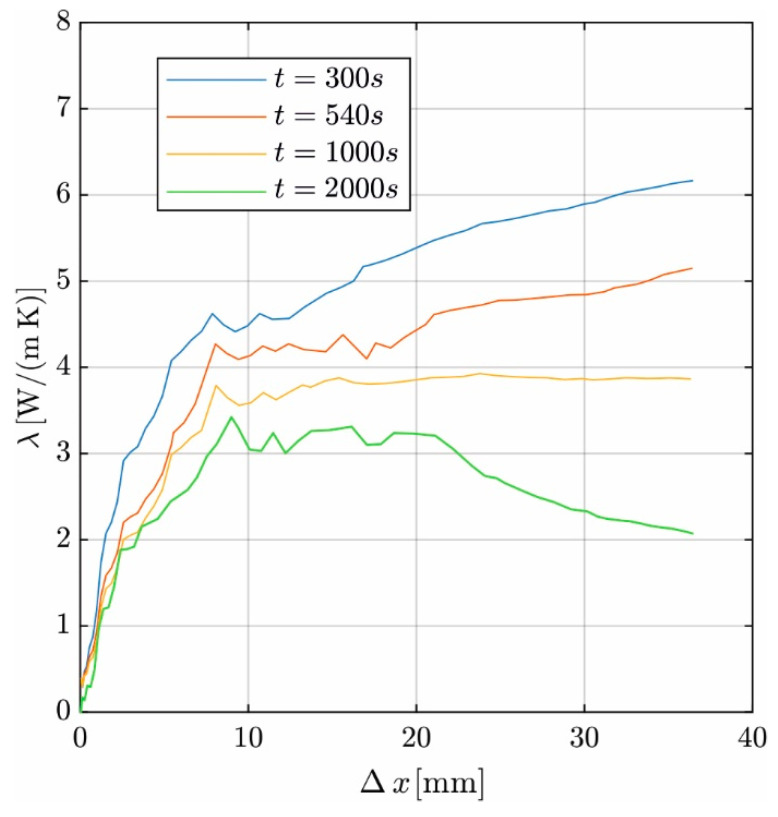
Effect of evaluation length on ***λ*** at different time points.

Figure 11 shows the effect of varying Δ*x* (abscissa) in the range between 1 mm and 38 mm on *λ* (ordinate) at different times. At all times, a plateau of stable results for *λ* is achieved, starting from Δ*x* = 9 mm. The length of the plateau increases as the tempering time increases. The curves rise for *t* = 300 s and *t* = 540 s as the temperature distribution is nonlinear along the evaluation length. For longer temper times, the curve decreases for Δ*x* > 20 mm due to the increasing impact of the hydration heat. For Δ*x* < 9 mm, the linearization is less robust against the measurement uncertainty and nonlinearity of the measured course due to the small number of measuring points. This causes the evaluated *λ*-values to decrease almost linearly.

The plateau defines the permissible Δ*x*. The results support the selection of Δ*x* = 9 mm in Section 3.4. For Δ*x* > 9 mm, the evaluation is robust against measurement uncertainties. On the other hand, a short evaluation length is more robust against the effects of hydration heat.

**Figure 12:**
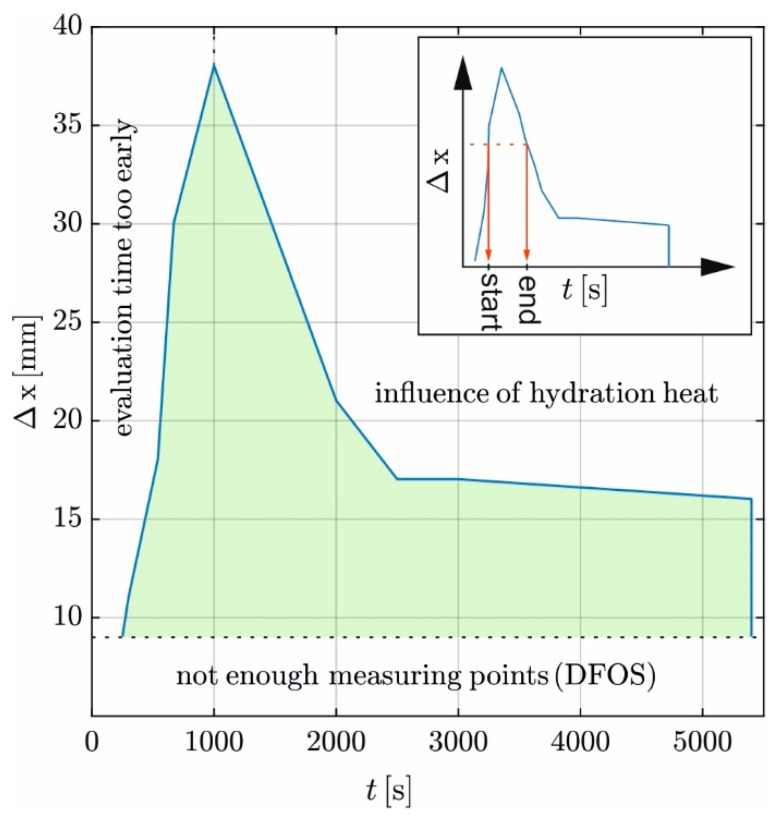
Relationship between evaluation length and the permissible evaluation interval.

This paper focuses on fine-grained concrete. The approach Δ*x* ≥ 3$D_{max}$ requires adjusting Δ*x* for normal concretes since $D_{max}$ is larger. Consequently, the permissible start time of evaluation shifts backward (see Figure 10). The permissible evaluation time intervals are given in Figure 12 by the green area. To determine the permissible evaluation time interval, a horizontal line is drawn from a chosen Δ*x*-value on the ordinate. From the intersection points with the boundaries of the green area, two vertical lines are drawn down to the abscissa, indicating the *start* and *end* of the evaluation time interval.

The boundaries of the permissible evaluation time follow from the minimum time to reach linearity of the temperature distribution along the evaluation length (left boundary) and the time when hydration effects become relevant to the temperature distribution (right boundary). So, as Δ*x* increases, the permissible evaluation time interval decreases. An evaluation length of Δ*x* ≥ 38 mm is not reasonable, as both boundaries overlap.

### 4.3. Influence of Induced Heat and Hydration Test

In the experiment, a heating mat with a two-point control was used to generate a temperature gradient in the concrete. After a certain time, the hydration heat superimposes the induced $\dot{Q}$. Therefore, the choice of heat source influences the method and the robustness of the results when deriving *λ*.

The used heating mat induces a nonconstant $\dot{Q}$ over time, and therefore a heat flux sensor is necessary to measure the time course (Figure 7). This also leads to an alternating course of ∆ϑ damped in time, which must be linearly smoothed to derive *λ*.

**Figure 13:**
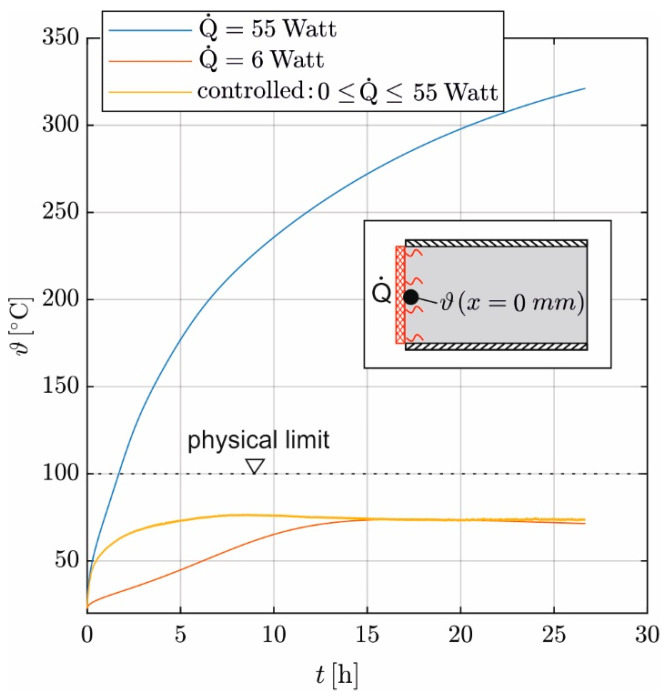
The temperature at the concrete surface with variations in induced heat according to the digital twin (Section 2.4).

One approach to simplify the evaluation is to induce a constant $\dot{Q}$. Figure 13 shows the calculated temperature development of the heating mat and, consequently, the concrete temperature at the top of the sample for constant $\dot{Q}$ values of 6 W and 55 W. The calculation is carried out using the digital twin described in Section 2.4, with 55 W corresponding to the maximum $\dot{Q}$ of the used heating mat and 6 W corresponding to the recorded mean $\dot{Q}$ in the stationary state to maintain the temperature constant at 80 °C. These are compared with the calculated temperature curve for a two-point controlled heat flux with ${\dot{Q}}_{max}$ = 55 W.

In the simulation with constant $\dot{Q}$ = 6 W, the heating mat needs approximately 15 h to reach the set temperature of 80 °C. In contrast, if a constant $\dot{Q}$ of 55 W is induced, the heating mat exceeds the critical temperature of 100 °C after approximately two hours. After that, the concrete continues to heat up even further. After 20 h, the calculated concrete temperature reaches over 300 °C. In the case of a controlled heat supply with ${\dot{Q}}_{max}$ = 55 W, the temperature curves follow the course of 55 W (constant) until a temperature of 48 °C is reached; $\dot{Q}$ is then continuously reduced so that the set temperature of 80 °C is not exceeded.

A low constant value of $\dot{Q}$ leads to a slow temperature increase in the concrete. As a result, it takes longer to achieve a linear temperature distribution on Δ*x*; the earliest permissible evaluation start is shifted backward. Additionally, lower concrete temperatures are more vulnerable to the impact of hydration heat, and smaller values of Δϑ reduce the robustness of the evaluation. On the other hand, a high constant $\dot{Q}$ leads to uncontrolled concrete heating—structural damage must be expected. Therefore, the control of $\dot{Q}$ is essential to heat the concrete quickly without exceeding the temperature limits.

**Figure 14:**
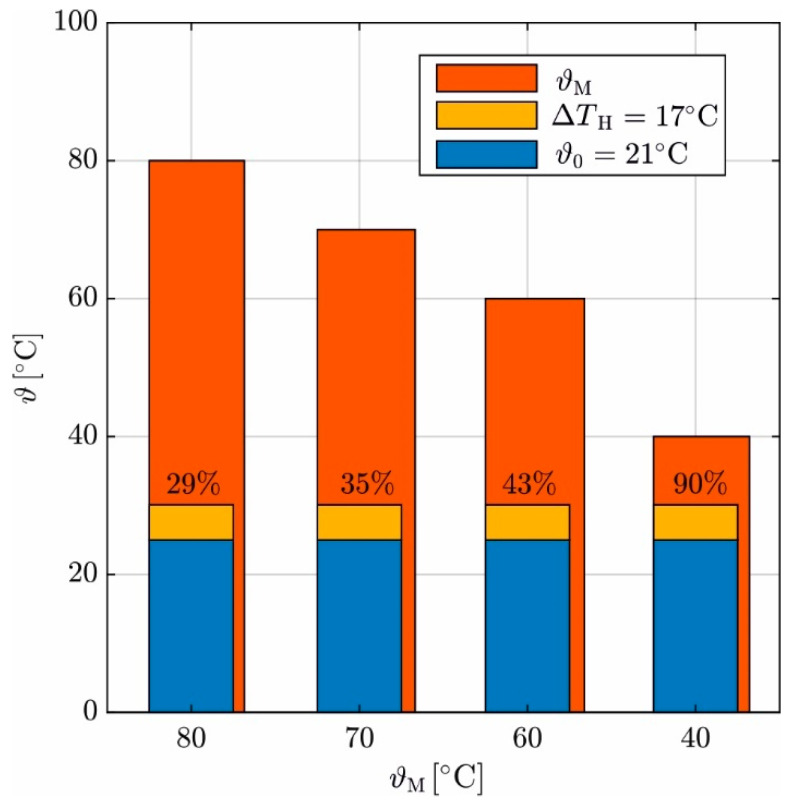
Hydration heat Δ*T*_H_ in relation to the externally supplied temperature $\vartheta_{M}$ minus the ambient temperature $\vartheta_{0}$.

The maximum concrete temperature results from the externally supplied heat plus the hydration heat and is limited to 100 °C. The increase in the temperature due to the hydration heat ${\Delta T}_{H}$ is approx. 17 °C for the investigated concrete and dimensions [36]. The heating mat reacts to the hydration heat with a delay due to the inertia of concrete heating and reduces $\dot{Q}$, so the concrete does not exceed the set temperature. Therefore, the maximum reached temperature in Figure 13 is only slightly above 80 °C. So, a temperature of the heating mat $\vartheta_{M}$ > 80 °C is also possible, e.g., 95 °C. The experimental work in [36] shows that a heat treatment accelerates hydration, but higher tempering does not increase ${\Delta T}_{H}$. Consequently, if $\vartheta_{M}$ decreases, the proportion of ${\Delta T}_{H}$ in concrete temperature increases. For $\vartheta_{M}$ = (80, 70, 60, 40) °C, Figure 14 shows the proportion of ${\Delta T}_{H}$ to ($\vartheta_{M}$*-*$\vartheta_{0}$); the ambient temperature $\vartheta_{0}$ = 21 °C is constant during the experiment. The relative share of ${\Delta T}_{H}$ to $\vartheta_{M}$ increases from 29% to 90% as $\vartheta_{M}$ is reduced.

The influence of different heating mat temperatures on the transient course of *λ* is analyzed with the digital twin (Section 2.4). The transient temperature distribution and the time series of $\dot{Q}$ are simulated for different heating mat temperatures $\vartheta_{M}$ = (80, 70, 60, 40) °C. Then, *λ* is derived according to Section 3.3.

**Figure 15:**
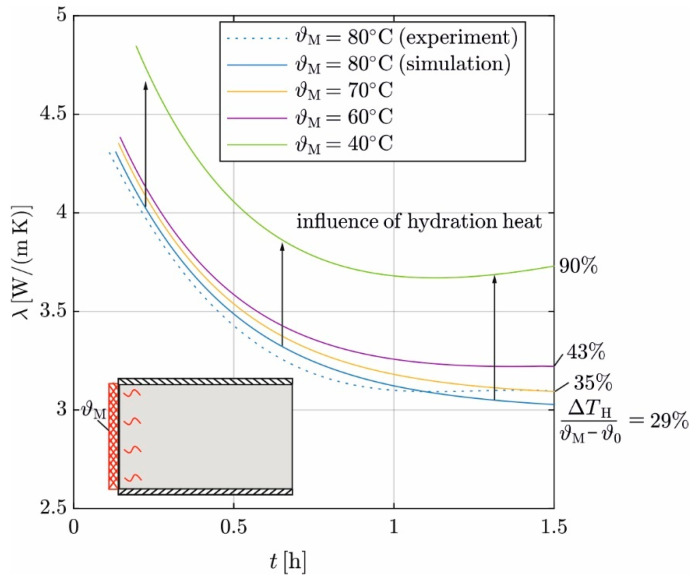
Influence of heating mat temperature $\vartheta_{M}$ on the transient evolution of *λ*.

Figure 15 shows the simulation results for *λ* and the results from the experiment. For $\vartheta_{M}$ = 80 °C, the determination coefficients between the experiment ($\lambda_{e})$ and simulation ($\lambda_{s})$ is *R*^2^ = 0.96. Reducing $\vartheta_{M}$ leads to an increase in the derived *λ* values; the curves shift upward. This can be attributed to the effects of hydration heat on Δϑ. $\dot{Q}$ between the heating mat and concrete exhibits delayed response to the additional heat from hydration and initially remains constant, resulting in a decrease in the derived *λ*. This effect increases as $\vartheta_{M}$ decreases.

A high heating mat temperature is thus less sensitive to the impact of hydration. Therefore, the heating mat temperature should be as high as possible without exceeding the limit of 100 °C. Therefore, 95 °C is a reasonable limit.

## 5. Conclusions

This paper presents a test rig for the experimental determination of the transient thermal conductivity coefficient of fine-grained concretes in the transition from liquid to solid. The test rig was exemplarily tested on a characteristic HPC. A digital twin of the test was used for further investigations of the setup and its boundary conditions. The following conclusions can be drawn:

- The thermal conductivity of concrete decreases in the transition from liquid to solid during hydration, following an exponentially decreasing course. For the investigated concrete, the thermal conductivity before hydration is *λ*_0_ ≈ 4.3 W/(mK). In the solid state, it is *λ*_hard_ ≈ 3.1 W/(mK); *λ*_hard_ is reached after about 1 h.
- In the transient state, only a short evaluation length can be considered for the temperature distribution. A minimum length must be maintained to reduce the influence of inhomogeneities in the concrete composition and measurement uncertainty. On the other hand, an extended evaluation length delimitates the permissible evaluation time and is more susceptible to the influence of hydration heat. Therefore, an evaluation length of three times the maximum grain diameter of the concrete is recommended.
- A high heating mat temperature reduces the relative influence of the released hydration heat. The concrete temperature must not exceed the critical temperature of 100 °C. The minimum heating mat temperature is 60 °C, and 95 °C is recommended under laboratory conditions.
- In order to achieve a linear temperature distribution over the evaluation length in a short time without exceeding the limit temperature of 100 °C, a controlled heat supply is necessary. In the experiment, a heating mat with a two-point control was used. The nonconstant-induced heat flux required using a heat flux sensor to measure the development over time. The derived *λ*-curve shows the oscillations of the heat flux and must be compensated by linearization. Using a more continuous control system such as a PID controller may be helpful to reduce the effect.

The developed test method is adaptable to a wide range of concrete compositions, even if the concrete is not suitable for heat treatment. The potential damage due to secondary ettringite formation is negligible for the derivation of *λ*. The method can also be extended to specimens of any size. The maximum grain size diameter of the tested concrete limits the application. A large maximum grain size diameter requires an increased test length, which increases the influence of disturbing variables such as the released hydration heat. The applicability of the test rig to non-fine-grained concretes needs to be investigated in further experiments.

## Figures and Tables

**Table 1:** Thermal conductivity coefficients of common concrete categories.

Concrete Category	Thermal Conductivity Coeff. *λ* [W/(mK)]
Aerated concrete	0.15–0.75
Normal concrete	1.15–1.65
High-performance concrete (HPC)	3.0
Ultra-high-performance concrete (UHPC)	6.0

**Table 2:** System parameters of the dig. twin.

Symbol	Value	Units
ρ	2440	kg/m^3^
$\lambda_{0}$	2.3	W/Km
η	2.05	ND ^1^
R	8.314	J/K
$Q_{x}$	$10^{8}$	J/m^3^
$\sigma_{1}$	16.1	W/(Km^2^)
$\sigma_{2}$	9.9	W/(Km^2^)
$c_{p}$	1320	J/(kgK)
$D_{m}$	$10^{9}$	m^2^/s
μ	56	1/s
E	35	kJ
$\bar{e}$	0.02	m/s
$T_{s,\;\infty }$	300	K
$m_{x}$	0.174	ND
$\theta_{a}$	0.01	ND

^1^ Nondimensional.

**Table 3:** Concrete composition of the HPC based on the binder Nanodur-Compound 5941.

Component	Type	Mass [kg/m^3^]
River Sand	0/2	426.0
Basalt	1/3	882.0
Binder	Nanodur-Compound 5941	1042.0
Water		159.8
Superplasticizer	Master Glenium ACE 430	12.3
Shrinkage reducer	Eclipse Floor	8.0
Hardening accelerator	Master X-Seed 100	12.3

**Table 4:** Comparison of the test results with literature data.

	Experiment	References [5,9,28,32]
*λ*_hard_ [W/mK]	≈3.1	≈3.0
*λ*_0_ [W/mK]	4.3 (*t* = 0.08 h)	3.9–4.7

## Data Availability

Data are contained within the article.
